# Expert consensus on irrigation and intracanal medication in root canal therapy

**DOI:** 10.1038/s41368-024-00280-5

**Published:** 2024-03-01

**Authors:** Xiaoying Zou, Xin Zheng, Yuhong Liang, Chengfei Zhang, Bing Fan, Jingping Liang, Junqi Ling, Zhuan Bian, Qing Yu, Benxiang Hou, Zhi Chen, Xi Wei, Lihong Qiu, Wenxia Chen, Wenxi He, Xin Xu, Liuyan Meng, Chen Zhang, Liming Chen, Shuli Deng, Yayan Lei, Xiaoli Xie, Xiaoyan Wang, Jinhua Yu, Jin Zhao, Song Shen, Xuedong Zhou, Lin Yue

**Affiliations:** 1 Department of Cariology and Endodontology, Peking University School and Hospital of Stomatology & National Center of Stomatology & National Clinical Research Center for Oral Diseases & National Engineering Laboratory for Digital and Material Technology of Stomatology & Beijing Key Laboratory of Digital Stomatology & Research Center of Engineering and Technology for Computerized Dentistry Ministry of Health & NMPA Key Laboratory for Dental Materials, Beijing, China; 2 Center of Stomatology, Peking University Hospital, Beijing, China; 3 State Key Laboratory of Oral Diseases & National Center for Stomatology & National Clinical Research Center for Oral Diseases & West China Hospital of Stomatology, Sichuan University, Chengdu, China; 4 Department of Emergency, Peking University School and Hospital of Stomatology & National Center of Stomatology & National Clinical Research Center for Oral Diseases & National Engineering Laboratory for Digital and Material Technology of Stomatology & Beijing Key Laboratory of Digital Stomatology & Research Center of Engineering and Technology for Computerized Dentistry Ministry of Health & NMPA Key Laboratory for Dental Materials, Beijing, China; 5 Restorative Dental Sciences, Endodontics, Faculty of Dentistry, The University of Hong Kong, Pok Fu Lam, Hong Kong SAR, China; 6 State Key Laboratory of Oral & Maxillofacial Reconstruction and Regeneration, Key Laboratory of Oral Biomedicine Ministry of Education, Hubei Key Laboratory of Stomatology, School & Hospital of Stomatology, Wuhan University, Wuhan, China; 7 Department of Endodontics, Shanghai Ninth People’s Hospital, Shanghai Jiao Tong University School of Medicine; College of Stomatology, Shanghai Jiao Tong University; National Clinical Research Center for Oral Diseases; National Center for Stomatology; Shanghai Key Laboratory of Stomatology, Shanghai, China; 8 Department of Operative Dentistry and Endodontics, Hospital of Stomatology, Guanghua School of Stomatology, Sun Yat-Sen University & Guangdong Provincial Key Laboratory of Stomatology , Guangzhou, China; 9 State Key Laboratory of Oral & Maxillofacial Reconstruction and Regeneration, National Clinical Research Center for Oral Diseases, Shaanxi Key Laboratory of Oral Diseases, Department of Operative Dentistry & Endodontics, School of Stomatology, The Fourth Military Medical University, Xián, China; 10 Center for Microscope Enhanced Dentistry, Beijing Stomatological Hospital, Capital Medical University, Beijing, China; 11 Department of Endodontics, School of Stomatology, China Medical University, Shenyang, China; 12 College & Hospital of Stomatology, Guangxi Medical University, Nanning, China; 13 Department of Stomatology, Air Force Medical Center, The Air Force Medical University, Beijing, China; 14 Department of Endodontics, Beijing Stomatological Hospital, School of Stomatology, Capital Medical University, Beijing, China; 15 Department of Endodontics, Guiyang Stomatological Hospital, Guiyang, China; 16 Stomatology Hospital, School of Stomatology, Zhejiang University School of Medicine, Zhejiang Provincial Clinical Research Center for Oral Diseases, Key Laboratory of Oral Biomedical Research of Zhejiang Province, Cancer Center of Zhejiang University, Engineering Research Center of Oral Biomaterials and Devices of Zhejiang Province, Hangzhou, China; 17 Department of Endodontics, the Affiliated Stomatological Hospital of Kunming Medical University, Kunming, China; 18 Department of Endodontology, Hunan Xiangya Stomatological Hospital, Central South University, Changsha, China; 19 Institute of Stomatology, Nanjing Medical University & Department of Endodontics, Affiliated Hospital of Stomatology, Nanjing Medical University, Nanjing, China; 20 Department of Endodontics, The First Affiliated Hospital of Xinjiang Medical University, Urumqi, China

**Keywords:** Oral diseases, Microbiology

## Abstract

Chemical cleaning and disinfection are crucial steps for eliminating infection in root canal treatment. However, irrigant selection or irrigation procedures are far from clear. The vapor lock effect in the apical region has yet to be solved, impeding irrigation efficacy and resulting in residual infections and compromised treatment outcomes. Additionally, ambiguous clinical indications for root canal medication and non-standardized dressing protocols must be clarified. Inappropriate intracanal medication may present side effects and jeopardize the therapeutic outcomes. Indeed, clinicians have been aware of these concerns for years. Based on the current evidence of studies, this article reviews the properties of various irrigants and intracanal medicaments and elucidates their effectiveness and interactions. The evolution of different kinetic irrigation methods, their effects, limitations, the paradigm shift, current indications, and effective operational procedures regarding intracanal medication are also discussed. This expert consensus aims to establish the clinical operation guidelines for root canal irrigation and a position statement on intracanal medication, thus facilitating a better understanding of infection control, standardizing clinical practice, and ultimately improving the success of endodontic therapy.

## Introduction

The core concept of root canal therapy is to control the infection in the root canal system by eradicating the existing infection and preventing any reinfection.^1^ However, the anatomical complexity of the root canal system and the diversity of root canal infections limit the efficacy of various strategies, such as mechanical instrumentation and irrigation, in eliminating root canal infections. For example, mechanical preparation by the movement of rotary files may not be able to follow the irregularities of the root canal wall, thus leaving the untouched area of the root canal surface up to more than 1/3–1/2.^2^ The anatomical factors of the root canal system and the limitations of instrumentation will undoubtedly lead to infection retention within the root canal.^3^ In literature, the complications of root canal treatment have been extensively discussed, and the effectiveness of mechanical preparation has been questioned, which was once overinterpreted.^4^ The infected root canal is a dead space favorable for bacterial growth and proliferation and a blind spot for host immunity due to the interruption of blood supply.^1^ There are three forms of infection in the canal system. (1) A mixture of suspended microorganisms and their metabolites, tissue debris and exudates, and foreign bodies. These infectious substances occupy the main, lateral, and accessory root canals, isthmus, and apical delta.^5^ (2) Microorganisms-formed biofilms adhered to the surface of the root canal wall, and various bacteria in the microenvironment, causing inflammation and even drug resistance.^6^ (3) Microorganisms and toxins enter in dentinal tubules of the root canal wall, with an invasion depth of 200–1 000 μm.^7–9^ These diversity of root canal infections raises the difficulty in root canal debridement. The consequence is that the residual microorganisms can sustain the infection state in the root canal, inducing persistent periapical tissue inflammation, and leading to root canal therapy failure.^10^

Mechanical preparation is fundamental in shaping the canal into a funnel and facilitating irrigation and obturation, but insufficient in canal cleaning and debridement. Thus, more endeavors have been made on chemical cleaning and disinfection, including root canal irrigation and intracanal medication. Chemical agents are used to inhibit or kill the remaining microorganisms in the infected root canal after mechanical preparation, especially in the wall of the root canal, the lateral accessory root canal, the isthmus, and the apical delta, which are complex anatomical regions that cannot be reached by instrumentation. Although various root canal irrigants and methodologies have been practiced in clinics, no single irrigant meets all the requirements for effective root canal cleaning safely and without side effects. The critical problem, vapor lock in the apical region, has yet to be solved either by conventional syringe irrigation or by various kinetic energy irrigation systems. There is no clear protocol or operation guideline for irrigation procedures in dental practice. Due to the ineffectiveness of root canal preparation, in the early 20th century, aldehydes and phenolics were advocated to seal the root canal to achieve the goal of disinfection.^11^ With technological advances and updated knowledge, modern root canal therapy recommends thorough root canal debridement by mechanical preparation and chemical irrigation. Root canal dressing agents have gradually faded, so the current medicaments and medication methods have changed considerably. Nevertheless, the clinical indications for root canal medication are unclear, and the dressing protocols are not standardized.

Based on the above-mentioned clinical concerns, there is an urgent need to refine the principles, objectives, medicament selection, operating instruments and techniques, and clinical procedures of root canal irrigation and medication and establish an expert consensus to better guide root canal infection control. This expert consensus aims to establish the clinical operation standards for root canal irrigation and medication based on the current evidence obtained from in vitro and in vivo studies.

## The pivotal role of root canal irrigation in modern endodontic therapy

Irrigation is regarded as an important part of root canal treatment. Irrigants are delivered into the root canal by needle or other tools during root canal instrumentation and irrigation. The irrigants can dissolve and remove infectious substances in the root canal, on the surface of the root canal wall, and within the dentinal tubules of the root canal wall. The chemicals contained in irrigation can kill or inhibit infectious microorganisms in the root canal, dissolve necrotic pulp tissue, neutralize toxins, remove the smear layer, and serve as lubricants.^12^

The efficacy of conventional syringe irrigation is affected by the depth the irrigation needle tips enter, the distance the irrigant penetrates apically to the needle, and the vapor lock effect.^13^ The effectiveness of conventional irrigation is often unsatisfactory. Thus, various instruments have been developed and used to activate the irrigants to maximize the debridement of complex root canal systems. In addition, more advanced techniques have been developed to augment physical kinetic energy to the liquid in the root canal by increasing the shear force on the root canal wall and activating the irrigants to improve the effect of chemical disinfection.

## Type of irrigants

### Current clinically used irrigants

#### Sodium hypochlorite (NaOCl)

The aqueous solution is a strong oxidant, which is alkaline and used as a disinfectant and household bleach. NaOCl is the most widely used root canal irrigation chemical due to its bactericidal and unique organic tissue-dissolving ability. NaOCl dissolves organic substances through three reactions: Degrading fatty acids into fatty acid salts and glycerol through saponification; Neutralization of amino acids to produce water and salt by neutralization reactions; Interference with microbial cell metabolism by chloramination of chlorine and amino groups.^14^ Since plaque biofilms, residual pulp tissue, and dentin are mainly organic tissues, NaOCl can exert a proficient tissue-dissolving effect on these tissues and improve the debridement effect of the unprepared areas. In addition, NaOCl has a broad antibacterial spectrum that can more effectively remove the infection in the root canal.^15^ Its main mode of action is hydrolyzation to form hypochlorous acid, which is further decomposed into new ecological oxygen to denigrate bacterial proteins, interfere with the oxidative phosphorylation of bacterial biofilm and the synthesis of bacterial DNA, and thus exerts a broad-spectrum bactericidal effect. The concentrations of NaOCl used in the clinic are 0.5%–8.25%. Over 2.5% NaOCl solution can dissolve organic tissues, and with the increase of concentration, temperature, volume, and time, the bactericidal effect and tissue dissolution ability are gradually boosted. At the same time, its irritation, causticity, and cytotoxicity to tissues are also increased. Root canal irrigation with NaOCl often follows root canal mechanical preparation and can be used as the final irrigation after instrumentation. A rubber dam must be placed during the treatment to protect the gingiva and oral mucosa. Extrusion of NaOCl solution from the apical foramen may cause periapical tissue damage, which has to be avoided.

#### Ethylenediamine tetraacetic acid (EDTA)

EDTA is a calcium chelator that can remove minerals from the smear layer and debris on the root canal wall. The smear layer not only hinders the contact between the chemical irrigants and the root canal wall but also provides a living environment for the growth of bacteria. Clinically, EDTA is often applied in combination with NaOCl solution (≥2.5%) to remove the smear layer on the root canal wall. NaOCl can dissolve organic components and eliminate bacteria. EDTA forms a complex with calcium ions in hydroxyapatite to dissolve inorganic components, such as dentin debris, thereby cleaning the root canal wall and opening the dentine tubules, facilitating the chemical molecules in the irrigant to penetrate the dentin tubules to exert an antibacterial effect in the deep locations. EDTA unceasingly softens the root canal wall and should not be used as a final irrigation.

#### Chlorhexidine (CHX)

CHX solution has a stable, long-acting, broad-spectrum antimicrobial property against Gram-positive and Gram-negative bacteria, and fungi. CHX in low concentration (0.2%) can enter the cell through the interaction with phospholipids on the surface of the cell membrane, increase the permeability of the cell wall, cause a large amount of potassium and phosphorus loss in the cell, and change the osmotic balance in and out of the cell. High concentration of chlorhexidine solution (2%) reacts directly with the cytoplasmic contents, resulting in bacterial death.^16^ As a cationic surfactant, CHX can be adsorbed on the surface of negatively charged substances in the cell wall of bacteria containing acidic proteins and can be slowly released by chelating with calcium ions in the root canal wall.^16^ On the other hand, its chelation with calcium ions in the root canal wall can slowly release the active ingredient of CHX,^17^ so that its bacteriostatic activity can be retained in the root canal system for up to 12 weeks,^18^ thus suppressing bacterial proliferation and exerting a long-term antibacterial effect.^15^ CHX is suitable for the final irrigation of severely infected root canals and retreatment cases.^12^ However, CHX has no tissue-dissolving ability and cannot remove the smear layer, so it cannot replace NaOCl and EDTA in clinical practice.

#### Ethanol

95% ethanol can be used as the final irrigation solution for root canals. Its strong volatility can quickly and effectively take away the water in the system and dry the root canal. It can also reduce the surface tension of the root canal wall, which is conducive to the sealer entering the complicated root canal structures and dentin tubules during the root canal filling process and improve the sealing effect.

#### Hydrogen peroxide

3% Hydrogen peroxide has was in root canal irrigation in history, but the evidence supporting its effectiveness is scarce. Its bactericidal effect on *Enterococcus faecalis* is weak.^19^ Thus, hydrogen peroxide is no longer recommended as a routine root canal irrigant.

### Interaction between irrigants

#### NaOCl and EDTA solution

The tissue-dissolving capacity of NaOCl decreases when combined with EDTA solution, with no free chlorine detected in the combinations.^20^

#### NaOCl and CHX

When combined, they react with each other to produce a brown precipitate containing para-chloraniline (PCA). The precipitate can block dentinal tubules and is difficult to remove.^21,22^ Therefore, the interaction should be avoided. In case NaOCl and CHX are used together, irrigation with water should be conducted to remove the residual NaOCl solution before CHX is used.

#### CHX and EDTA

Although CHX solution does not react chemically with EDTA solution when mixed, it forms a salt that is insoluble in water and appears as a white precipitate.^23^

## Development of clinical root canal irrigation methods and techniques

### Conventional syringe irrigation

A 5 mL syringe is the most commonly used instrument for root canal irrigation. With this syringe, the flow rate can reach at least 0.20–0.25 mL/s,^24^ and the irrigant penetration can be 1–2 mm apically to the needle tip within the root canal. The needles have various specifications, mainly differing in the presence of an open or closed end, the diameter, and the outlet numbers. The recommended diameters are 30 gauge needles (0.298–0.320 mm outside diameter, corresponding to a 30# file). There are two main types of needle end openings: open-ended (flat/ bevelled/ notched) and close-ended (side-vented/ double side-vented/ multi-vented).^25^ The needle with the lateral opening is more conducive to the coronal reflux of the irrigant, which can effectively reduce the amount of irrigant pushed out beyond the apical foramen.^26^ Key points and precautions of syringe irrigation are:

- Fill the pulp chamber and root canal with irrigants before the first step of root canal treatment, always keeping the root canal system under irrigant immersion.
- Frequent and extensive root canal irrigation should be performed during mechanical preparation, with 2 mL of irrigant per canal between file replacements.
- A total of 10–20 mL of irrigant is used for each root canal during the whole mechanical preparation.
- The needle should be inserted as deep into the root canal as possible at 2 mm from the working length. It should not be inserted too tightly, which may cause poor reflux and the irrigant and debris extrusion beyond the apical foramen.
- The needle should be moved longitudinally in the canal with up and down motion in a small range, and gently push the syringe plunger. Do not apply excessive force apically.^27^
- The syringe should have a Luer Lock threaded fitting to avoid the needle falling off and the irrigants splashing due to excessive pressure during irrigation, which may cause skin injuries or the patient clothes bleaching.

The efficacy of conventional syringe irrigation is limited, depending on needle insertion depth into the root canal, the diameter and shape of the needle, and the root canal width, curvature, and taper.^28,29^ Because the root canals are closed-ended cavities, the air bubbles entrapped at the apical part of the root canal can block irrigant penetration in this area, called the *vapor lock* phenomenon.^30^ Due to the vapor lock effect, sufficient infection debridement in the apical region is impossible using conventional syringe irrigation alone.

### Mechanical agitation irrigation

In order to enhance the irrigant penetration and refreshment in the apical part of the root canal, a gutta-percha or a file matching the root canal size is proposed in clinical practice. The vapor lock effect can be disrupted by manual agitation so that fresh irrigants can enter the apical segment of the root canal and improve the cleaning effect.^13^ Due to the low efficiency of manual agitation, motorized agitation instruments and equipment have emerged. Most of them are made of NiTi alloy with excellent elasticity. The instruments inserted into the root canal are driven by a powered motor to rotate continuously to improve the flushing effect of the liquid flow. The specially designed spoon-shaped instruments can also take advantage of their extensibility to expand and deform during movement to adapt to the irregular shape of the root canal and even directly touch the canal wall to remove the adhesions on the canal wall.^31^ At present, such devices include XP-endo Finisher, M3-Max, etc. The XP-endo Finisher was found to be superior to conventional syringe irrigation in removing the smear layer, dentin debris, and bacterial biofilm in the root canal.^32,33^ Another agitation instrument is the Finisher GF brush, a final agitation file in the Gentlefile system made of six strands of stainless-steel flexible wires. Under the centrifugal effect produced by 6500 rpm high-speed rotation, the wires are spread out to whip, scrape and smooth the root canal wall, and activate irrigants by mechanical agitation,^34,35^ thus improving the root canal debridement effect of sodium hypochlorite.^36^ The Gentlefile system’s unique design can produce a “tornado” effect, which can guide the irrigants into the apical part and suck the liquid and debris back to the orifice direction, providing new ideas and methods to solve the irrigation challenges. The problem with mechanical agitation is that irrigants may extrude the apical foramen and cause some postoperative pain.^37,38^

### Physical energizing irrigation

Physical kinetic energy can be applied to the irrigants in the root canal to change the flow pattern, increase the flushing intensity, improve the wall shear stress, and activate the chemical composition of the irrigants, which can promote the irrigants to enter the complex of the root canal system and further exert the biochemical effects. It’s also known as kinetic energy irrigation. The physical kinetic energy includes ultrasonic and sonic energy, positive and negative pressure, laser, etc. How liquid energy is converted by various physical kinetic energy and its effects differ.

#### Ultrasonic irrigation

Ultrasonics was first introduced in endodontics by Richman.^39^ The range of frequencies of the ultrasonic device was between 25 000 and 40 000 Hz.^40^ Under ultrasound activation, the irrigants form a circular or vortex-like motion that rolls rapidly, which is the effect of acoustic streaming.^41^ The shear stress generated by acoustic streaming along the root canal wall facilitates the removal of tissue and biofilm attached to the root canal wall and suspended debris and bacteria in the canal. In addition, the bubbles in the liquid caused by the acoustic streaming continue to grow and become unstable, eventually collapse in a violent implosion. The strong shock wave and instantaneous high flow rate generated by the explosion of bubbles are conducive to the removal of infectious substances, which is known as the cavitation effect.^41^ The early ultrasonic irrigation method is active ultrasonic irrigation (AUI). In the process of irrigation, the ultrasonic file acts on the root canal wall and cuts the dentin, which may cause damage to the root canal wall.^42^ On the other hand, because the ultrasonic file attaches to the root canal wall, the acoustic streaming effect cannot be generated, which may limit the effectiveness of ultrasonic irrigation. The concept of Passive Ultrasonic Irrigation (PUI) was first proposed by Weller et al. in 1980, defined as the “non-cutting” movement form of the ultrasonic file.^43^ It means that the ultrasonic file does not touch the root canal wall, so it does not remove it, thus avoiding the problems of AUI mentioned above.^41^ PUI only transfers energy through the vibration of the ultrasonic file into the liquid and utilizes significant acoustic streaming and cavitation effects to achieve debridement.^44^ Clinically, a 15–25# ultrasonic file or a special ultrasonic tip without a cutting edge is placed in the root canal 1–2 mm short from the working length, which can clean the apical part well.^45^ Still, irrigants may be extruded beyond the apical foramen if the ultrasonic file is too close to the apex. Additionally, the ultrasonic file is suggested to be placed above the curvature of the root canal to avoid excessive contact with the root canal wall, perforation, or instrument separation.^46^

There are two common modes of PUI, namely continuous and intermittent irrigation. With continuous irrigation, the irrigant is continuously flowing to irrigate the root canal simultaneously during ultrasonic activation. The other method was to inject irrigants into the root canal, then insert an ultrasonic file, and intermittently irrigate the static irrigant in the root canal. Each root canal was ultrasonically activated three times, 20 s each time, for a total of 1 min. After each PUI, 2 mL of the canal was irrigated with a syringe, and the irrigant was refreshed.

#### Sonic irrigation

Tronstad et al. first reported the sonic devices in endodontic treatment.^47^ The sound waves generated mechanical vibration of the liquid in the root canal, broke the vapor lock effect at the root apex through the acoustic streaming and made the irrigating fluid smoothly enter the apical area to achieve root canal cleaning. The sonic activation has a lower driving frequency but a greater amplitude than ultrasound devices. The tips used in the current sonic irrigation equipment are made of soft polymeric materials and can be inserted into the middle or lower segments of the curved root canal.^48^ Studies have shown that when the ultrasonic tip is constrained by the root canal wall, the amplitude will be significantly reduced, and its acoustic streaming will be significantly weakened. However, if constrained, the sonic tip can still produce longitudinal vibration with large amplitudes.^49^ In addition, acoustic streaming can be generated along the working tip’s length, and the acoustic streaming’s attenuation degree is smaller than that of ultrasound. Therefore, although the acoustic streaming effect produced by sonic irrigation is weaker than that of ultrasonic irrigation in theory, a desired irrigation effect may still be obtained in practice.^18^ The first-generation EndoActivator, which appeared in 2010, is a low-frequency sonic device that operates at 160 Hz, 175 Hz, and 190 Hz.^50^ Li et al. compared the smear layer cleaning effect of ultrasound and EndoActivator on the root canal wall.^51^ It was shown that the first-generation EndoActivator achieved a smear layer cleaning effect similar to ultrasound in the middle and upper root canal segments. It was better than ultrasound in the apical part. However, the bacterial inhibition ability in the deep dentinal tubules of EndoActivator was not as good as that of PUI.^51^ In order to improve the acoustic streaming effect, the second generation EndoActivator appeared in 2023, which increased the vibration frequency to 300 Hz. However, the high-frequency sonic device EDDY, which came out in 2015, has a frequency of 5 000–6 000 Hz. Studies have shown that EDDY has a better debridement effect on dentin debris and smear layer than low-frequency sonic device (EndoActivator).^52^ Liu et al. showed that Eddy could achieve a similar antibacterial effect in dentin tubules as ultrasound in the middle and upper root canals.^53^ However, neither high-frequency nor low-frequency sonic activation showed a good effect on removing the bacteria in the root canal, especially in the deep dentinal tubules of the apical root canal wall.

#### Negative pressure irrigation

The combination of positive-pressure irrigation and negative-pressure suction in the root canal allowed the irrigants to reach the apical region without extruding the apical foramen.^54^ The representative product is EndoVac. The working tip of the EndoVac was not allowed to enter the apical part until the canal was prepared to 35#,^55^ and its effect of irrigation in the apical area is better than that of the traditional positive pressure irrigation.^56^ Launched in 2015, the GentleWave is designed to clean and disinfect complex root canal systems using multi-frequency sound waves to form enriched cavitation microbubbles and broad-spectrum sound fields. The pulp chamber sealing device should be used to create a positive pressure flushing and negative pressure suction environment after the specially designed working head enters the pulp chamber, and the irrigant in the pulp chamber can be sucked out while pushing it into the root canal at the same time. The negative pressure formed at the apex avoids the extrusion of the irrigant into the apical foramen.^57^ The GentleWave system can also improve the organic tissue solubilization of sodium hypochlorite,^58^ and its bacterial biofilm removal effect was better than that of ultrasound in the central root canal and isthmus.^59^

#### Laser activated irrigation

The use of lasers in endodontic treatment began in 1971. With the development of optical fiber transmission systems, laser has been widely used in endodontics in the 1990s. The erbium (Er) family laser is considered the most suitable type for laser activation irrigation, including Er: YAG laser (2940 nm) and Er, Cr: YSGG laser (2780 nm). Erbium laser has a high affinity for water and hydroxyapatite, which can produce a strong activation effect and shock wave during irrigation. Fast flow and high shear stress were induced on the root canal wall. Laser can also produce reactive oxygen to destroy biofilms and directly accelerate bacterial death. The most significant feature of the Erbium laser is its remote bactericidal efficacy. However, the root canal’s complex and variable anatomical structure will gradually decrease its optomechanical effectiveness from the coronal to the apical part. Photon-initiated photoacoustic streaming (PIPS) is another laser activation irrigation system developed from the traditional erbium laser. The principle of its application in root canal irrigation is to transmit low energy (20–50 mJ) with an extremely short pulse (50 μs), which generates a sustained and intense shock wave, causing violent movement of the irrigant in the root canal.^60^ Studies have shown that PIPS can promote irrigant penetration into dentinal tubules,^61^ and achieve a better disinfection effect than ultrasound.^62^ However, in vitro studies have shown that a small amount of irrigant may extrude the apical foramen,^63^ it is still unclear whether it will cause clinical postoperative pain. In addition, laser-activated irrigation is not widely used in clinical practice because it requires expensive laser equipment.

## Recommended clinical procedures of root canal irrigation in root canal treatment

Root canal irrigation should be performed under rubber dam conditions and a microscope. Rubber dams can separate the operation area from the internal environment of the oral cavity, avoid contamination of the operation area, improve the operation efficiency, and prevent complications such as the irritation of the oral mucosa by flushing irrigants and the accidental ingestion of irrigants.^1,64^ Using a microscope can help the clinician better observe the movement of the irrigating solution in the root canal and accurately judge the cleanliness of the root canal. The basic principle of root canal irrigation strategy is irrigation before starting instrumentation, frequent and abundant irrigation, and irrigation throughout the treatment. The following procedures are recommended.

- After entering the pulp chamber, NaOCl solution is injected first to fill in the pulp chamber and root canal, then identification of orifice locations and root canal negotiation were performed.
- During access cavity and root canal mechanical preparation, copious NaOCl irrigation is recommended until the instrumentation is completed.
- Irrigation with 2.50%–5.25% NaOCl solution (2 mL/root canal) between each file is recommended during the instrumentation process.^65^
- After the mechanical instrumentation, the irrigant amount for final irrigation should be more than 5 mL/root canal. The apical size of the infected root canal is generally suggested to be prepared to at least 30# in order for a 30 G irrigation needle to reach sufficient depth and ensure effective action of irrigants in the apical third region, as well as achieve optimal removal of pulp tissue, debris, and infectious substances in the apical region.^25,66,67^
- The root canal should be irrigated before medication or obturation. The sequence of final irrigation is as follows: NaOCl (2.50%–5.25%) for 1 min, EDTA (17%) for 1 min to remove the smear layer, and final NaOCl is reintroduced in the root canal system for 30 s to penetrate further into the opened dentinal tubules that now have been cleared of smear layer to inhibit the bacteria.^25^ To ensure the irrigant’s penetration into the root canal, the chemical irrigant can be activated by kinetic energy irrigation, such as ultrasonic, sonic, laser, negative pressure irrigation, or mechanical agitation. The protocol is as follows: The irrigant is refreshed three times in each root canal, and ultrasonic irrigation is performed for 20 s within the irrigant filled in the pulp chamber and root canal system for 1 minute to increase the effect of the chemical irritant. Pay attention to the water irrigation between the two irrigants to avoid mutual reactions.
- For severely infected root canals, especially those with sinus tracts or purulence or retreatment cases, 2% chlorhexidine can be used as the final irrigant. However, NaOCl or EDTA in the root canal should be replaced with water first.^24,68^
- Before root canal obturation, 95% ethanol can be used to rinse the root canal, with 3 mL/root canal. With the rapid evaporation of ethanol, water can be taken away to obtain the drying effect of the whole root canal system, which is conducive to the entry and attachment of filling materials.

Combining multiple irrigants is recommended to achieve complementary or enhanced root canal cleaning. At the same time, attention should be paid to the incompatibility and interaction of chemical agents.

## Attitude toward intracanal medication

The primary objective of intracanal medication is to eliminate microbial and toxin burden within the root canal after mechanical preparation and irrigation. Introduction of antimicrobial and disinfecting chemical agents into the root canal can directly impede or eradicate microorganisms, neutralize toxins, modulate environmental pH, and create favorable biological conditions for periapical tissue repair and regeneration.^69,70^

From 1891 to the early 20th century, due to limited resources and inadequate understanding of root canal mechanical preparation and irrigation techniques, scholars endeavored to achieve root canal disinfection by encapsulating potent volatile drugs like formaldehyde and phenol within the root canal. Commonly employed root canal disinfectants included formaldehyde phenol, camphor para-chlorophenol, camphor phenol, etc.^71,72^ During this period, multiple medication applications were necessary in conjunction with sampling and cultivation within the canals until bacterial testing results indicated sterility prior to root canal obturation.^73^ However, using intracanal medicaments alone is insufficient for optimal therapeutic outcomes without adequate root canal preparation, as revealed by numerous studies.^74,75^ Furthermore, the medications mentioned above exhibit potent cytotoxicity and poor biocompatibility. They can induce damage to periodontal fibers and impede periapical tissue healing,^74^ potentially leading to systemic allergies in severe cases.^11^ Therefore, their utilization is currently not recommended in clinical practice.^65,76–78^ Over the past 30 years, advancements in root canal cleaning and sealing techniques have led to more effective infection control within the root canal. Consequently, the demand for intracanal medication has gradually decreased. It is now recognized that interappointment medication may augment effectiveness while increasing the risk of reinfection between visits. According to clinical studies and systematic analysis on non-infected root canals, there is no statistically significant difference in effectiveness between single-visit and multiple-visit root canal therapy when evaluating indicators, such as periapical bone density,^79^ healing rate of lesions,^80^ and postoperative pain.^81^ Therefore, using intracanal medicaments for non-infected root canals is not recommended. However, it is still advisable to use appropriate medications to manage symptoms, control infection, and evaluate prognosis in cases of severe root canal infections.^82–85^ These drugs should prioritize biocompatibility, stable physicochemical properties, and degradability. Highly bactericidal pastes are recommended as they require direct contact with the root canal walls to create a physical barrier sealing off the pulp cavity.

## Types of intracanal medicaments

The ideal intracanal medicaments should have the following characteristics: (1) Strong antimicrobial abilities, neutralizing toxins, and sustained disinfection capability. (2) Permeability and flowability. (3) Formation of a physical-chemical barrier within the root canal. (4) Excellent biocompatibility, reducing inflammation in periapical tissues without causing additional irritation to the apical tissue. (5) Not interfering with the repair, induction of healing and hard tissue formation in periapical tissues. (6) Easy removal. Currently, available drugs in clinical practice have not yet met all these requirements.

### Calcium hydroxide (Ca(OH)_2_)

It is an extensively researched and widely utilized disinfectant for root canals, exhibiting potent antibacterial activity by releasing hydroxyl ions in water to create an alkaline environment. It exerts bactericidal effects through cell membrane disruption, protein denaturation, and DNA damage. Moreover, it effectively neutralizes bacterial endotoxins on the root canal walls and promotes tissue healing by counteracting acidic substances generated during inflammation.^86,87^ Clinical studies have demonstrated that calcium hydroxide can significantly reduce the number of cultivable bacteria within the root canal.^88,89^ Additionally, it promotes mineralized tissue formation and facilitates the repair of periapical hard tissues. It is commonly utilized as an intracanal medicament for treating periapical lesions in immaturely developed teeth and preventing/treating inflammatory root resorption.^87,90–92^ Nevertheless, the limitations of calcium hydroxide as an intracanal medication are primarily due to (1) poor antibacterial effects against specific pathogens like *E. faecalis* and *Candida albicans*;^93,94^ (2) dentin’s ability to buffer the high pH environment produced by calcium hydroxide, affecting its antibacterial activity in vitro;^95^ (3) limited volatility of calcium hydroxide,^96^ making it challenging to target microorganisms in areas such as apical deltas and isthmuses;^97,98^ (4) slow onset of action and removal difficulty.^87^ Studies have shown inconsistent results regarding the impact of calcium hydroxide paste on tooth fracture resistance.^99,100^ Some research indicates that prolonged application of calcium hydroxide paste to root canal walls can reduce dentin’s three-point bending strength and fracture resistance, possibly due to its strong alkalinity and water solubility leading to the loss of organic and inorganic components in dentin.^99,100^ According to reports, oil-based calcium hydroxide (such as Vitapex) has a slow release. It does not significantly impact tooth fracture resistance,^99^ making it suitable for relatively long-term (2–4 weeks) use. Calcium hydroxide remains the preferred agent for intracanal medication at present.

Calcium hydroxide is powdered and can be formulated into various types of intracanal medication by combining it with different solvents. An ideal formulation should possess good flowability and permeability while not affecting or promoting the dissociation of calcium hydroxide ions, as its action depends on pH value and direct contact. Solvents are classified into aqueous, viscous, and oily based on their viscosity and ability to facilitate calcium hydroxide dissociation. Among them, water-based solvents are commonly used. Calcium hydroxide powder is typically mixed with physiological saline to form a paste, or pre-made water-based calcium hydroxide can be used for better flowability and ion release capability. An oil-based calcium hydroxide medication like Vitapex may be chosen if long-term medication is required.^99^ However, doubts exist regarding the disinfection efficacy of viscous or oily calcium hydroxide formulations due to their significant inhibition of hydroxyl ion dissociation and release. Calcium hydroxide is commonly combined with radiopaque agents such as barium sulfate, bismuth carbonate, iodoform, etc., to enhance its X-ray opacity,^101,102^ facilitating the evaluation of intracanal medication quality using X-ray images.

### Chlorhexidine (CHX)

It belongs to the category of cationic antimicrobial agents and exhibits a broad-spectrum antibacterial activity. The commonly employed clinical formulation is 0.2%–2.0% chlorhexidine gluconate gel, which can be utilized independently or in conjunction with calcium hydroxide.^12^ However, the efficacy of chlorhexidine as a standalone intracanal medication remains uncertain. In vitro studies have demonstrated that chlorhexidine exhibits superior antibacterial efficacy compared to calcium hydroxide, effectively eliminating *E. faecalis* biofilm at a concentration of 2%.^103^ In vivo investigations have revealed that chlorhexidine reduces bacterial counts within the root canal and prevents colonization when used as an intracanal medication for 1 week.^104^ However, clinical trials have reported no statistically significant difference in antibacterial effects between chlorhexidine and calcium hydroxide after one or two weeks of intracanal application. Meanwhile, the capacity of chlorhexidine to reduce endotoxin levels within the root canal is weaker than calcium hydroxide.^105^ There is still controversy regarding the effectiveness of combining chlorhexidine with calcium hydroxide, and the results of various studies are inconsistent. In vitro experiments have shown that this combination effectively eliminates *E. faecalis*, surpassing the antibacterial effects of calcium hydroxide alone,^106^ and it can also reduce bacterial types and quantities in initially infected root canals.^107^ However, clinical trials have found no statistically significant difference in antibacterial effects between using these two substances or using only calcium hydroxide.^108^ There is insufficient evidence to suggest that chlorhexidine used alone or in combination with calcium hydroxide has superior effects compared to calcium hydroxide alone.

### Antibiotics

Throughout the history of root canal treatment, antibiotics used as intracanal medicaments have attracted attention several times. However, to this day, they have not become mainstream due to their insufficient effectiveness and the potential for bacteria resistance caused by antibiotics. Triple antibiotic paste (TAP), consisting of metronidazole, minocycline, and ciprofloxacin, is an effective antibiotic formulation. These antibiotics complement each other in terms of antimicrobial efficacy, providing a broad spectrum of antibacterial activity with deep penetration and long-lasting effects. In vitro studies have demonstrated that TAP exhibits superior microbial clearance against infected dental pulp compared to calcium hydroxide and 2% chlorhexidine gel.^109^ TAP is commonly used in regenerative endodontic treatment,^110^ typically applied for two weeks. Its use in conventional root canal therapy has not been observed. The drawbacks of TAP include (1) Minocycline can cause tooth discoloration; (2) Complete removal from the root canal is challenging; (3) It only effectively targets metabolically active microorganisms; (4) Antibiotic resistance may occur. Therefore, in 2016, the European Society of Endodontology statement still recommends using calcium hydroxide as an intracanal medication in regenerative endodontic treatment.

### Corticosteroids

The glucocorticoids, as steroid hormones, possess anti-inflammatory and anti-allergic effects. They can reduce the release of inflammatory mediators, decrease capillary permeability, alleviate edema and exudation, and relieve inflammation in periapical tissues.^111^ The use of corticosteroids should be limited to the minimum effective dose and shortest duration in order to achieve treatment goals while minimizing adverse reactions in multiple body systems, including cardiovascular, digestive, hematologic, endocrine, and immune systems.^112^ In root canal therapy, corticosteroids are commonly combined with antibiotics to effectively reduce swelling and pain caused by acute apical periodontitis and postoperative discomfort.^113^ Currently available corticosteroid-antibiotic pastes include Septomixine, Pulpomixine, and Ledermix paste. Septomixine and Pulpomixine contain neomycin and neomycin B, respectively, but they have limited antibacterial activity against bacteria causing root canal infections.^114^ On the other hand, Ledermix paste contains 1% triamcinolone acetonide and 3% demeclocycline, which has anti-inflammatory effects that help reduce root resorption and promote apical healing.^115,116^ The use of steroids for intracanal medicaments is limited due to their side effects, including immunosuppression,^117^ tooth discoloration,^118^ and drug interactions.^119^

## Indications and non-indications for intracanal medication

### Indications for calcium hydroxide paste as an intracanal medication

- Severe root canal infection: clinical signs of severe root canal infection include a sinus tract, active exudation, swelling, pain to palpation and percussion, and extensive radiological periapical tissue lesions.

- Can not complete root canal cleaning, shaping, and obturation in a single visit: Patients may require treatment to be completed in multiple visits due to various systemic and local factors, including but not limited to systemic diseases (such as diabetes and cardiovascular diseases), advanced age with reduced treatment tolerance, restricted mouth opening, temporomandibular joint disorders, or oral and maxillofacial injuries.
- Observing infection control’s effectiveness during treatment is crucial for evaluating prognosis and determining treatment plans. Intracanal medicaments offer a short-term window to assess changes in symptoms and judge the success of infection control, providing valuable information for clinical diagnosis and treatment strategies.
- In teeth with internal or external resorption, intending to perform treatment for rescuing the tooth.
- In immature permanent teeth with open apices, plan to perform apexification or regenerative endodontic treatment.

### Non-indications for intracanal medication

- For non-infected root canals, completing the root canal therapy in a single visit is advocated.
- In the case of adult permanent teeth with periapical lesions and root resorption leading to an open apex, intend to perform apical barrier treatment.

## Clinical protocol for using calcium hydroxide as an intracanal medication in root canal treatment

- Perform final irrigation and dry the root canal thoroughly.
- Radiopaque calcium hydroxide paste is recommended for facilitating X-ray examination. To ensure easy removal of medicaments from the root canal, it is preferable to use water-based agents. The medication should be precisely injected or introduced throughout the entire final working length (FWL) of the root canal to achieve maximal disinfection.
- Using glass ionomer cement (GIC) to seal the crown entrance.
- Immediate X-ray imaging is suggested, to examine the quality of intracanal medicaments, which provides a predictive reference for controlling infection and guiding communication with patients about treatment procedures and prognosis.
- The sealing period for root canal therapy is 1–2 weeks.^65,120^
- Medicament removal: Water-based calcium hydroxide can be removed by irrigation, and it is recommended to use dynamic irrigation under microscope.^44,121^ Irrigation needle with bristle brush (NaviTip FX), XP-endoFinisher, M3-Max, and Finisher GF Brush can be used for agitation during root canal irrigation,^32–34,122^ which enhances the effectiveness and efficiency of removing medicaments.
- In general, intracanal medication is typically performed 1–2 times during interappointment visits.^12^ If symptoms and signs persist, a thorough analysis should be conducted to identify any deficiencies in the root canal cleaning process or the presence of extra-radicular infections, other diseases, or issues.^123,124^ This analysis will help develop targeted infection control strategies for subsequent steps or consider modifying the treatment plan. It is advised to consider an alternative treatment plan rather than repeating the same medication for more than three visits.^125,126^

## Consequences and treatment of intracanal medicament extrusion

Extrusion of calcium hydroxide beyond the apical foramen can induce inflammation in periapical tissues, developing iatrogenic or chemical apical periodontitis, ultimately failing root canal therapy.^127^ However, research has also demonstrated that incorporating radiopaque agents influences the impact of calcium hydroxide on the healing process of periapical lesions beyond the apical foramen.^128^ Using a paste containing barium sulfate affects the regeneration of periapical tissues, potentially due to an immune response triggered by barium sulfate,^129^ and its promotion of osteoclast differentiation leading to bone resorption.^130^ Furthermore, a case report study suggests that the extrusion of calcium hydroxide does not influence the healing of periapical lesions.^131^ Calcium hydroxide paste without radiopaque agents can be completely absorbed, while paste with added barium sulfate cannot even after complete tissue healing in periapical regions.^131^ Existing research on the consequences and outcomes of intracanal medication extrusion remains insufficient. Therefore, when applying calcium hydroxide, intentionally exceeding the apical foramen should be avoided.

In cases where calcium hydroxide exceeds the apical foramen, treatment options include (1) regular monitoring through X-rays and clinical examination to evaluate periapical healing and medication absorption; (2) administration of anti-inflammatory or analgesic drugs to alleviate pain or swelling; (3) if persistent symptoms, infection, or damage to adjacent structures (such as maxillary sinuses or mandibular nerve canal) occur, endodontic microsurgery should be performed for cleaning purposes.

## Position statement on intracanal medication in modern endodontic therapy

This article presents a statement on intracanal medication in contemporary endodontic therapy:

- For teeth with vital pulp where the root canals are not infected, the primary focus of root canal therapy is to strictly follow the infection control principles: meticulous sterilization of instruments and materials, use of a rubber dam for isolation during treatment, thoroughly cleaning the root canals, and completely obturating the root canal system. Intracanal medication is not essential, and completing root canal treatment in a single visit is encouraged.
- For infected root canals with periapical lesions caused by pulp infection and necrosis and cases of root canal retreatments, especially those with extensive apical lesions, severe swelling, and pain, presence of sinus tracts, or active exudation, intracanal medication is needed to reduce bacteria and toxin load in the root canal system. The interappointment period of intracanal medication provides a window of opportunity for the assessment of the effect of infection control.
- The intracanal medicaments should consist of a radiopaque agent and fill the entire root canal system to establish a robust physical barrier. Additionally, it should tightly seal the coronal access to prevent any potential leakage from the crown. Immediate evaluation of the intracanal medication by X-ray imaging is suggested.

Overall, adequate mechanical preparation and irrigation are the primary measures for controlling root canal infection during root canal therapy, while intracanal medication is a supplementary approach.

In summary, chemical irrigation is essential in infection control, and medication is supportive for persistent infection, especially teeth with sinus tract, during root canal therapy. The irrigation and medication protocols for apexification or regenerative endodontic procedures should refer to the corresponding expert consensus.^110^ Given the intricate nature of root canal anatomy and the diverse range of infections encountered, it is imperative to develop safer and more effective irrigants, innovate more practical and feasible operating techniques and procedures, and develop smaller and more affordable equipment for root canal irrigation. With advancements in techniques for cleaning root canals, the focus on intracanal medicaments is becoming secondary. The emergence of next-generation antimicrobial peptides, nanoparticles, and other drugs/formulations may bring about fundamental changes in intracanal medication and revolutionize the procedures and concepts underlying root canal therapy. Nonetheless, the ultimate objective of root canal therapy remains unaltered: controlling infection to preserve the affected tooth maximally.
